# Cul3-KLHL20 ubiquitin ligase: physiological functions, stress responses, and disease implications

**DOI:** 10.1186/s13008-016-0017-2

**Published:** 2016-04-01

**Authors:** Hsin-Yi Chen, Chin-Chih Liu, Ruey-Hwa Chen

**Affiliations:** Graduate Institute of Cancer Biology and Drug Discovery, College of Medical Science and Technology, Taipei Medical University, Taipei, Taiwan; Institute of Biological Chemistry, Academia Sinica, Taipei, Taiwan; Institute of Biochemical Sciences, College of Life Science, National Taiwan University, Taipei, Taiwan

**Keywords:** Cul3 ubiquitin ligases, KLHL20, Ubiquitination, Hypoxia, Angiogenesis, Cancer, Autophagy, Actin cytoskeleton

## Abstract

Cullin-RING ubiquitin ligases are the largest Ubiquitin ligase family in eukaryotes and are multi-protein complexes. In these complexes, the Cullin protein serves as a scaffold to connect two functional modules of the ligases, the catalytic subunit and substrate-binding subunit. KLHL20 is a substrate-binding subunit of Cullin3 (Cul3) ubiquitin ligase. Recent studies have identified a number of substrates of KLHL20-based ubiquitin ligase. Through ubiquitination of these substrates, KLHL20 elicits diverse cellular functions, some of which are associated with human diseases. Furthermore, the functions, subcellular localizations, and expression of KLHL20 are regulated by several physiological and stressed signals, which allow KLHL20 to preferentially act on certain substrates to response to these signals. Here, we provide a summary of the functions and regulations of KLHL20 in several physiological processes and stress responses and its disease implications.

## Background

Protein ubiquitination regulates a broad range of physiological processes and disease conditions. Originally identified as a mechanism that targets protein for degradation by the 26S proteasome [1, 2], ubiquitination is now appreciated as the most versatile posttranslational modification given the ability to conjugate structurally and functionally distinct ubiquitin polymer to the substrate [3]. A critical step in the protein ubiquitination involves ubiquitin ligase, which transfers the ubiquitin moiety to the substrate to confer substrate specificity [4]. Cullin-RING multi-protein complexes comprise the largest family of ubiquitin ligases, in which one particular Cullin serves as a scaffold for linking two functional modules: the catalytic RING finger protein Rbx1 or Roc2 and the substrate-binding module for bringing substrate within the proximity to the catalytic module [5]. In the Cullin3 (Cul3) family of ubiquitin ligases, the Bric-a-brac/tramtrack/broad complex (BTB) domain-containing protein functions as the substrate adaptor to bridge Cul3 and substrate, which is in analogous to the Skp1-F-box heterodimer in the Cullin1 complex [6, 7]. The human genome contains nearly 200 BTB proteins [8], suggesting the existence of large numbers of distinct Cul3 complexes.

The BTB domain, also known as BTB/POZ domain, was originally identified as a protein–protein interaction domain [9]. Most BTB family proteins contain one or two other domains and therefore can be classified into several subfamilies. Among them, the kelch-repeat domain subfamily is most prevalent in metazoan [8]. The human genome contains more than 50 BTB-kelch members, which are termed as Kelch-like 1-42 (KLHL1-42) and kelch and BTB domain containing 1-14 (KBTBD1-14) with the KLHL proteins containing an additional BACK domain in between the BTB and kelch domain [10, 11]. In BTB-kelch proteins, the BTB domain adopts a five α-helical fold resembling other Cullin-binding proteins in the Cullin-RING ligase complexes, such as Skp1 and ElonginC [8]. In addition, a 3-box motif in the proximal part of BACK domain (also known as IVR domain) also contributes to the interaction with Cul3 [10, 12]. This explains why certain BTB-domain proteins lacking the 3-box region, such as BTBD12, are not co-purified with Cul3 from cells [12]. The most C-terminus of these proteins contains the kelch-repeat domain which forms a β-propeller structure for substrate interaction [13].

**Fig. 1:**
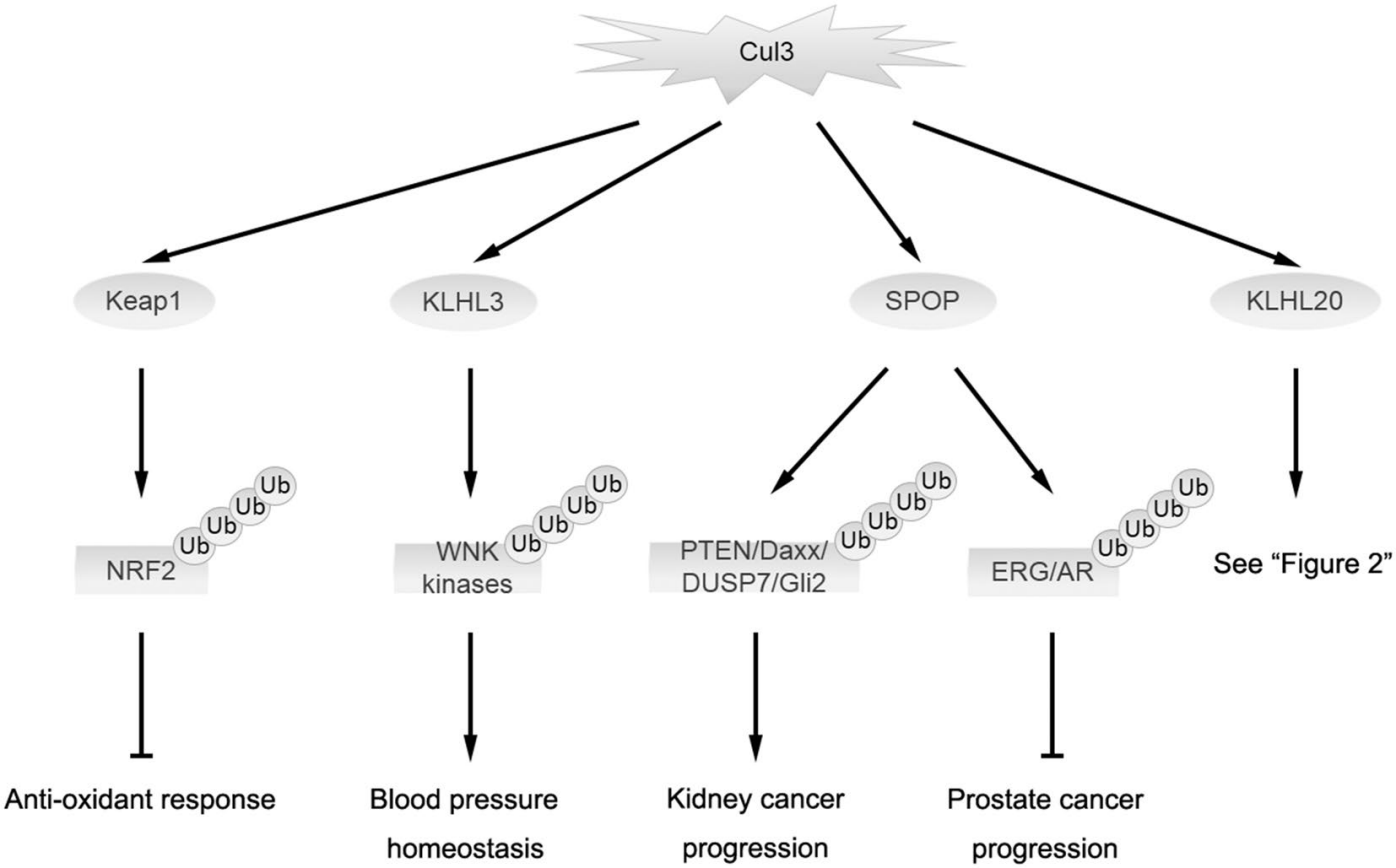
Overview of certain important targets and functions of Cul3 ubiquitin ligases

**Fig. 2:**
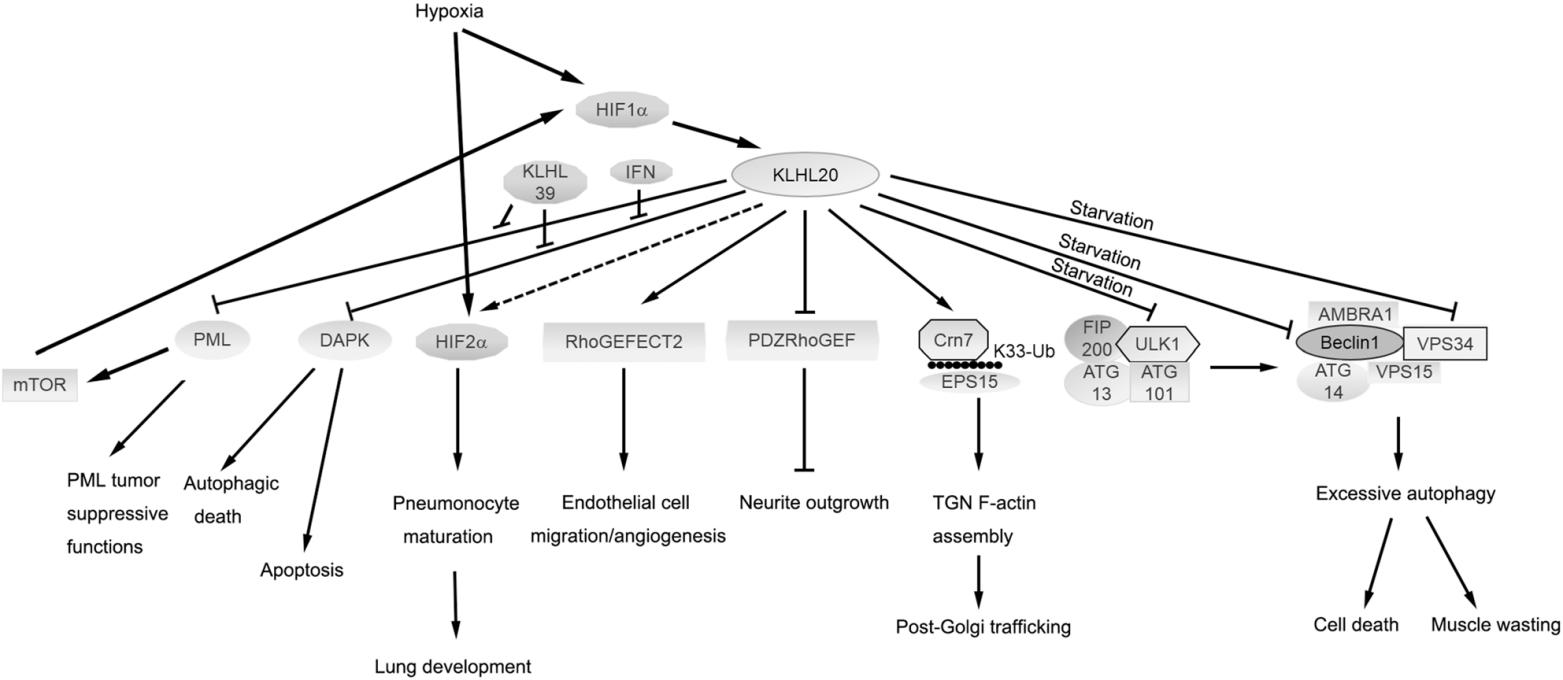
Overview of the regulators, effectors and biological functions of KLHL20

To date, many Cul3 substrate adaptors have been discovered and their ubiquitination plays pivotal roles in various cellular processes and disease states (Fig. 1). For instance, the prototype of BTB-kelch protein Keap1 (designated as KLHL19) mediates the ubiquitin-dependent degradation of Nrf2, a master regulator of anti-oxidant responses [14, 15]. KLHL3, another BTB-kelch protein, targets WNK kinases for degradation which is important for blood pressure homeostasis [16, 17]. SPOP, a BTB/POZ-containing protein, plays context-dependent roles in tumorigenesis, i.e., suppressing prostate cancer by inducing the destruction of androgen receptor and ERG oncoprotein and promoting kidney cancers through the degradation of tumor suppressor proteins PTEN, ERK phosphatases, Daxx, and Gli2 [18–21]. KLHL20 was discovered before the identification of a functional link of BTB-kelch proteins to Cul3 ubiquitin ligases. This 64-kDa protein was discovered by its recruitment to the adherent junction of epithelial cells, implicating an involvement in cell–cell adhesion [22]. Furthermore, KLHL20 is induced by Wnt inhibitor DKK1 in keratinocytes and in the MelanoDerm of skin [23]. Subsequent studies indicated that KLHL20 forms a ubiquitin ligase complex with Cul3 and Rbx1 [24]. To date, a number of substrates of this complex have been identified, which collectively point to its versatile functions. This review will provide an overview of the functions of KLHL20 in several cellular processes and disease states and its regulation under various physiological and stressed conditions (Fig. 2). Finally, we will highlight a number of key questions that remain to be answered.

## Regulation of cell death

The first substrate identified for Cul3-KLHL20 ubiquitin ligase is death-associated protein kinase (DAPK). Originally discovered as a mediator of interferon-γ (IFN-γ)-induced cell death [25], DAPK is subsequently found to function as a positive regulator of apoptosis in response to a wide range of stimuli, such as TNF-α, Fas [26], TGF-β [27], ceramide [28], oncogene activation [29], unligated netrin receptor [30, 31], matrix detachment [32], ER stress [33], and neurotoxicity [34], and can also regulate autophagic cell death and programmed necrosis [35–37]. In addition to promoting cell death, DAPK elicits other anti-cancer functions, such as suppression of metastasis by regulating cell migration, adhesion, and cytoskeleton remodeling [32, 38–40] and inhibition of cell transformation by inactivating the phospho-Ser/Thr directed isomerase Pin1 [41]. In line with these tumor suppressive functions, DAPK expression or activity is frequently downregulated in various types of human cancers through hypermethylation- or miRNA-induced gene silencing or posttranslational modification-induced inactivation [42–45]. The linkage of DAPK to KLHL20 was first identified through a yeast two-hybrid screen in which the death domain of DAPK is capable of binding a C-terminal portion of KLHL20. Further analysis revealed that KLHL20 interacts with DAPK in mammalian cells under physiological conditions. Furthermore, KLHL20 utilizes its kelch-repeat domain to recruit DAPK to Cul3. The Cul3-KLHL20 ubiquitin ligase promotes DAPK polyubiquitination and proteasomal degradation. Through such a mechanism, KLHL20 elicits an anti-apoptotic function by counteracting DAPK-induced apoptosis [24].

Interestingly, the Cul3-KLHL20-mediated DAPK ubiquitination and degradation is blocked in response to IFN-α or IFN-γ treatment, which provides an explanation for an important role of DAPK in apoptotic and autophagic death induced by these stimuli. Mechanistically, this regulation of DAPK ubiquitination is attributed to a subcellular redistribution of KLHL20. Under IFN-α/γ-treated conditions, KLHL20 is enriched in a nuclear subdomain called PML-nuclear body, thereby separating KLHL20 from the cytoplasm/actin cytoskeleton-localized DAPK. The redistribution of KLHL20 to PML-nuclear bodies is presumably triggered by IFN-α/γ-induced transcription of PML [46, 47], as PML competes with DAPK for binding KLHL20 [24]. In support of this notion, in certain multiple myeloma cells where IFN-α/γ fail to induce PML and PML-nuclear bodies, KLHL20 retains in the cytoplasm and perinuclear region, thus allowing a persistent ubiquitination and degradation of DAPK. Importantly, such persistent DAPK destabilization accounts for one mechanism by which tumor cells are resistant to IFN-α/γ treatment [24]. Thus, KLHL20 is capable of controlling the effectiveness of IFN-based anti-cancer therapy through destabilizing the pro-death factor DAPK.

## Suppression of PML tumor suppressive functions

The findings of a localization of KLHL20 in PML-nuclear bodies and its interaction with PML under IFN-α/γ-treated conditions imply a link of KLHL20 to PML. The *PML* (promyelocytic leukemia) gene was originally identified based on its localization at the break point of the *t*(15:17) chromosome translocation found in most cases of acute promyelocytic leukemia [48]. The PML protein is essential for the formation of PML-nuclear bodies [49] and elicits pleiotropic anti-tumor effects, such as suppression of proliferation, neoangiogenesis, cell migration, and promotion of apoptosis and senescence [50–53]. Additionally, PML regulates cancer cell metabolism and suppresses cancer stem cell maintenance [54, 55]. Consistent with these tumor suppressive functions, the expression of PML protein, but not its mRNA, is frequently lost or reduced in a wide range of human malignancies, such as colon, lung, prostate, breast, brain tumors, germ cell tumors, and non-Hodgkin’s lymphoma [56]. Evidence has emerged that ubiquitin-mediated proteasomal degradation is a key mechanism for PML degradation in tumors [57]. The Cul3-KLHL20 complex has been demonstrated to mediate PML polyubiquitination, leading to the downregulation of PML protein and PML-nuclear bodies [58]. However, targeting PML to KLHL20 requires two consecutive posttranslational modifications on PML, that is, phosphorylation at S518 by CDK1/2 followed by Pin1-mediated prolylisomerization of the peptide bond between S518 and P519. This finding indicates a cell cycle-dependent regulation of PML stability, which is consistent with the highest PML protein level in the G1 phase [59]. Since CDK1/2 activity and Pin1 expression are frequently upregulated in tumors, KLHL20-dependent PML ubiquitination and degradation is expected to be enhanced in tumors. Furthermore, KLHL20 is expected to elicit certain oncogenic roles through blocking the tumor suppressive effects of PML. Indeed, KLHL20 confers tumor-promoting functions, such as transformation, migration, and survival, which are dependent on PML downregulation [58].

## Participation in tumor hypoxia responses and angiogenesis

An additional layer of the regulation of KLHL20-mediated PML ubiquitination comes from the identification of *KLHL20* as a transcriptional target of hypoxia-inducible factor-1 (HIF-1) [58]. HIF-1 and its paralog HIF-2 are key molecules to mediate the adaptation of hypoxia by transcriptional activation of a large panel of genes containing “hypoxia responsive element” (HRE) on their promoters [60]. The promoter of *KLHL20* contains two HREs which are both involved in hypoxia-induced transactivation. Consistent with the induction of KLHL20 by HIF-1, ubiquitination and degradation of PML is potentiated in hypoxia conditions through a HIF-1- and KLHL20-dependent manner [58]. Reciprocally, PML negatively regulates the translation of HIF-1α through a mechanism involving mTOR deactivation [50]. The counter-inhibitory circuit between PML and HIF-1 indicates that KLHL20-mediated PML ubiquitination and degradation participates in a double- negative feedback regulation to potentiate HIF-1α accumulation in hypoxia conditions. In support of this hypothesis, KLHL20 knockdown significantly attenuates hypoxia-induced HIF-1α and HIF-2α [58]. Thus, the KLHL20/PML pathway not only downregulates tumor suppressor PML but also is part of a feedback control mechanism for a robust induction of HIF-1 and HIF-2 in response to hypoxia. HIF-1- and HIF-2-regulated transcriptional networks play a crucial role in many aspects of tumor biology, such as immortalization, autocrine growth, metabolic reprogramming, cancer stem cell maintenance, invasion, metastasis, tumor angiogenesis, and resistance to therapy [61]. These effects collectively contribute to the aggressiveness of diseases and are termed as tumor hypoxia responses.

Importantly, KLHL20 is demonstrated to act as a positive regulator of various tumor hypoxia responses through PML degradation. Consistent with these tumor-promoting functions, aberrant expression of several key molecules of the KLHL20/PML pathway, such as HIF-1α [62], Pin1 [63], and PML [56], has been reported in many types of cancers. In human prostate cancer, high expression of HIF-1α, KLHL20, and Pin1 is frequent observed and correlates with PML low expression [58]. More importantly, patients displaying the signature of high HIF-1α, high KLHL20, high Pin1, and low PML expression pattern are found to be progressively increased with disease progression. These clinical findings support the significance of KLHL20/PML pathway in the progression of prostate cancer and suggest a promise for targeting this pathway in the treatment of aggressive prostate cancers.

In addition to regulating HIF-1α and HIF-2α indirectly through PML degradation, KLHL20 can also bind HIF-2α [64]. This study also showed a positive role of KLHL20 in regulating HIF-2α protein expression and HIF-2α-mediated gene expression through a hypoxia- and VHL-independent mechanisms, even though it remains undetermined whether KLHL20-HIF-2α interaction contributes to these effects.

The important role of KLHL20 in hypoxia signaling is not confined to tumor cells. Indeed, KLHL20 is preferentially expressed in endothelial cells where it is induced by hypoxia. Depletion of KLHL20 in endothelial cells impairs VEGF- and FGF-induced migration and sprouting angiogenesis without affecting cell proliferation [65]. Mechanistic studies indicated that KLHL20 binds to RhoGEF ECT2 to control VEGF-induced RhoA activation. Taken together, KLHL20 can not only amplify tumor hypoxia responses to stimulate angiogenesis in tumor microenvironments but also function directly in endothelial cells to promote their migration and sprouting angiogenesis.

## Regulation of actin cytoskeletons and vesicular trafficking

The nomenclature of kelch domain was originated from *Drosophilakelch*, whose mutation results in a disorganized F-actin structure in the ring canals which connect oocyte with accessory cells [66]. Similar to kelch, many kelch domain proteins are known to bind and regulate actin cytoskeletons through their kelch domain [66]. KLHL20 is also able to bind F-actin in vitro and colocalizes with F-actin at cell–cell contact sites in vivo [22]. Although it remains unclear whether KLHL20 acts directly on actin cytoskeleton to modulate its architecture and dynamics, a recent study indicates that KLHL20 promotes the assembly of a particular pool of F-actin by catalyzing the ubiquitination of an actin-binding protein [67]. This study was initiated by the observation of a predominant localization of KLHL20 in a perinuclear region coincident with the markers of Golgi apparatus, especially with the marker of Trans-Golgi network (TGN). Remarkably, the TGN distribution of KLHL20 is disrupted by brefeldin A, which blocks the activation of Arf- or Arl-family GTPases. The Arf/Arl-dependent recruitment of KLHL20 to TGN implies a role of KLHL20 in Golgi-mediated intracellular trafficking. Indeed, KLHL20 is essential for the anterograde transport of several cargoes from TGN to plasma membrane or endosomes but is dispensable for retrograde transport. This function of KLHL20 is mediated by its involvement in the formation and elongation of post-Golgi carrier tubules [67], which are subsequently cleaved to generate transport vesicles [68].

Because the function of KLHL20 in anterograde transport is dependent on its interaction with Cul3, ubiquitination of a Cul3-KLHL20 substrate should be responsible for this trafficking function. Among the KLHL20-interacting proteins, coronin 7 (Crn7) is also involved in post-Golgi trafficking and functions in a step similar to KLHL20 in this process [69]. Biochemical analyses indicated that Crn7 is a *bona fide* substrate of Cul3-KLHL20 ubiquitin ligase [67]. However, unlike other substrates of this ubiquitin ligase, KLHL20 cannot influence on the stability of Crn7 but promotes an atypical K33-linked polyubiquitination on Crn7. This ubiquitination facilitates the recruitment of Crn7 to TGN by binding to a ubiquitin-binding protein Eps15, which is translocated to TGN prior to Crn7 during post-Golgi trafficking. How would TGN recruitment of Crn7 contribute to trafficking? Crn7 is an F-actin-binding protein and can stabilize F-actin filaments [70]. Short F-actin filaments are assembled around Golgi apparatus where they are critical for the biogenesis of post-Golgi carriers [68, 71, 72]. Indeed, the polymerization of F-actin, in conjunction with myosin motors, exerts mechanical force to assist membrane deformation, leading to the formation of tubule carriers. Accordingly, blockage of K33-ubiquitin chain formation, Crn7 ubiquitination, or Crn7-Eps15 interaction each impairs the assembly of TGN-pool F-actin [67]. Conversely, enforced targeting of Crn7 to TGN bypasses the requirement of its K33-linked ubiquitination for TGN-pool F-actin assembly and anterograde trafficking. Thus, KLHL20 controls post-Golgi transport by mediating a K33-polyubiquitination on the actin-regulating protein Crn7.

## Regulation of neural morphogenesis

The influence of KLHL20 on cytoskeletons is not confined to its action on Crn7. The Rho-family GTPase RhoA activates multiple effectors to control the dynamics of actin and microtubules and RhoA activity is positively regulated by a family of guanine nucleotide exchange factors (RhoGFEs) [73]. The connection of KLHL20 to RhoGEF was discovered by studying its function in primary neurons. In both hippocampal and cortical neurons, KLHL20 promotes neurite outgrowth and arborization and these effects require its interaction with Cul3 [74]. Subsequent analysis identified the brain-enriched PDZ-RhoGEF as an interactor of KLHL20 and a substrate of Cul3-KLHL20 ubiquitin ligase. KLHL20-mediated ubiquitination of PDZ-RhoGEF leads to its proteasomal degradation and consequently the inactivation of RhoA. Restriction of RhoA activity in turn facilitates the spreading of axonal growth cone and promotes neurite outgrowth. Importantly, the inhibitory effect of KLHL20 depletion in neurite outgrowth is rescued by depletion of PDZ-RhoGEF, indicating the critical function of KLHL20/PDZ-RhoGEF axis in regulating neural morphogenesis during differentiation.

Neurite outgrowth and arborization are crucial for the establishment of connections during the development and remodeling of nervous system. Neurotrophins regulate a number of important functions in vertebrate nervous system including survival, differentiation, axon outgrowth, and myelination [75]. The physiological significance of KLHL20/PDZ-RhoGEF axis is evidenced by its involvement in neurotrophin-induced neurite outgrowth [74]. It turns out that a p38 MAPK-induced phosphorylation on PDZ-RhoGEF is required for its recruitment to KLHL20. Neurotrophins, such as neurotrophin-3 and brain-derived neurotrophic factor, induce the activation of p38 MAPK [76], thereby resulting in a persistent degradation of PDZ-RhoGEF by KLHL20. In keeping with this mode of PDZ-RhoGEF regulation by neurotrophins, KLHL20 depletion or p38 inactivation blocks neurotrophin-induced neurite outgrowth. Furthermore, depletion of PDZ-RhoGEF rescues the effect of p38 inhibitor on neurotrophin-induced neurite outgrowth. However, in the absence of p38 inhibitor, PDZ-RhoGEF siRNA does not show any significant effect, consistent with its persistent degradation under this situation. Thus, stimulation of PDZ-RhoGEF degradation through KLHL20-dependent ubiquitination represents a mechanism for neurotrophin-induced neurite outgrowth, which indicates a key role of KLHL20/PDZ-RhoGEF axis in the morphogenesis of neural circuits.

## Control of autophagy termination

The linkage between KLHL20 and autophagy was recently uncovered [77]. Autophagy is a lysosome-dependent catabolic process which is critical for the maintenance of cellular homeostasis when cells encounter various stressed conditions. The autophagic process consists of several steps, in which ULK1 and VPS34 complexes play essential roles in autophagy initiation and autophagosome nucleation, respectively [78, 79]. ULK1 is a serine/threonine kinase and forms a functional complex with ATG13, FIP200, and ATG101. ULK1 is activated in response to autophagic stresses, such as starvation [80–82]. The activated ULK1 in turn relays signals to VPS34 complex, which comprises of the phosphatidylinositol 3-kinase VPS34 and regulatory subunits VPS15, Beclin-1, and ATG14 [83]. The activated VPS34 complex is recruited to phagophores, the sites of autophagosome formation, in an ULK1- and ATG14-dependent manner [84, 85]. At phagophores, VPS34 generates PtdIns3P, which recruits other factors for autophagosome biogenesis [86, 87]. Autophagy is a dynamic and self-limiting process, which is gradually turned off after its activation [88].

The study unveiling a critical role of KLHL20 in autophagy termination was initiated by the identification of ULK1 as an interacting protein of the kelch-repeat domain of KLHL20 [77]. Subsequent studies found that KLHL20-based Ubiquitin ligase promotes ULK1 ubiquitination and proteasomal degradation. Notably, the interaction between KLHL20 and ULK1 is induced by starvation. Further biochemical analysis revealed that KLHL20 targets the Ser1042/Thr1046-autophosphorylated ULK1 for polyubiquitination. This mechanism constitutes a feedback regulation to specifically remove ULK1 that is activated by the autophagic process. In addition to ULK1, KLHL20 also controls autophagy-dependent degradation of VPS34 and Beclin-1. This function is mediated by autophagy-induced recruitment of KLHL20 to phagophores, where it colocalizes with Belcin-1 and VPS34 to facilitate their K48-linked ubiquitination. The degradation of ULK1, VPS34, and Beclin-1 further leads to the degradation of their complex components ATG13 and ATG14. Collectively, KLHL20 coordinates the degradation of multiple components of ULK1 and VPS34 complexes through a direct or indirect mechanism. The continuous degradation of these autophagy-initiating factors gradually leads to their exhaustion in the prolonged phase of stressed conditions, and thus contributes to autophagy termination. Accordingly, depletion of KLHL20 or impairment of ULK1 autophosphorylation results in a persistent and excessive autophagy activity even at the prolonged starvation. Thus, KLHL20 functions as a master regulator of autophagy termination.

What is the physiological significance of KLHL20-mediated autophagy termination? At a cellular level, blockage of KLHL20-dependent autophagy regulation or impairment of ULK1 autophosphorylation increases cell death under prolonged starvation, indicating that autophagy termination prevents uncontrolled cellular degradation to maintain cell survival. At an organism level, skeletal muscle specific-KLHL20 knockout mice exhibit an aggravation of muscle atrophy in an experimental diabetes model and this effect is dependent on excessive autophagy activity. Thus, KLHL20-dependent autophagy termination is important in maintaining tissue homeostasis by preventing excessive muscle wasting under this disease state.

## Lessons from *KLHL20* knockout mice

Given the multiple functions of KLHL20, it is conceivable that >50 % of the *KLHL20* knockout mice exhibit neonatal lethality [89, 90]. This neonatal death is associated with respiratory failure, reduced lung aeration, and increased septum thickness [90]. In addition, surfactant expression is significantly reduced, indicating a defect in the maturation of type II pneumocytes. Despite these defects, KLHL20 expression is absent in lung epithelium but present in lung endothelial cells and pericytes. Furthermore, *KLHL20*^−*/*−^ lung exhibit enhanced apoptosis and regressive vascularization. These findings suggest that KLHL20 potentiates a paracrine function of lung endothelium to influence on the maturation of lung epithelium. Mechanistically, expression of HIF-2α and VEGF are significantly reduced in *KLHL20* deficient lung. Given the importance of HIF-2α and VEGF in late-stage maturation of type II pneumocytes [91–93], the KLHL20/HIF-2α axis likely underlines the lung development defect seen in *KLHL20*^−*/*−^ mice.

The *KLHL20*^−*/*−^ mice that can survive beyond the neonatal stage show compensatory elevation of HIF-2α and VEGF expression for an unknown reason [90]. However, they display progressive corneal neovascular dystrophy starting as early as week 3 [89]. The pathology is initiated by hyperplasia of the corneal epithelium, followed by conversion of non-keratinized corneal epithelium to superficially keratinized cells, and eventually leading to total corneal opacity. The abnormalities seen in the cornea of *KLHL20*^−*/*−^ mice are likely due to a decreased corneal epithelial integrity, consistent with the recruitment of KLHL20 to the cell–cell adhesion sites seen in cultured epithelial cells [22]. During disease progression, massive corneal vascularization is also observed, which is associated with increased expression of angiopoietin-1 and downregulation of miR-204, a negative regulator of angiopoietin-1 [94]. However, given the positive role of KLHL20 in angiogenesis in other systems, the corneal vascularization seen in *KLHL20*^−*/*−^ mice is likely a secondary effect induced by high proliferation activity and scarification of the corneal epithelium.

The neonatal lethality of *KLHL20*^−*/*−^ mice may hinder the analysis of its pathophysiological functions in the adulthood. This limitation is alleviated by the establishment of *KLHL20*^*flox/flox*^ mice which enable the study of KLHL20 function in a tissue-specific manner [77].

## Regulation of KLHL20

### Regulation of KLHL20 subcellular distributions

The pleiotropic functions of KLHL20 are consistent with its diverse and variable subcellular localizations. In the normal culturing conditions, KLHL20 is predominantly distributed to a perinuclear region where it is colocalized with markers of cis-Golgi, trans-Golgi, and TGN [67]. Quantitative analysis indicated that KLHL20 is most precisely colocalized with TGN marker. In addition to the perinuclear region, KLHL20 immunofluorescent signal is weakly detected in cytoplasm, nucleoplasm, PML-nuclear bodies, and cell–cell contact region. As described above, KLHL20 subcellular localizations can be modulated under several physiological settings and stressed conditions. In response to autophagy induction, KLHL20 relocates to vesicle-like structures which partially overlap with the nascent phagophores marked by ATG16L [77]. Although ATG9 also travels from TGN to phagophore during autophagic process, KLHL20 vesicles do not overlap with ATG9 vesicles (Chin-Chih Liu and Ruey-Hwa Chen, unpublished results). The nature of KLHL20 vesicles and the mechanism of KLHL20 redistribution in response to autophagy are currently unknown.

Notably, even though KLHL20 is enriched in the Golgi apparatus, this distribution is not static and is dependent on the activity of Arf/Arl-family proteins [67]. This indicates that Golgi-mediated intracellular trafficking facilitates a Golgi distribution of KLHL20. Likewise, KLHL20 is transiently recruited to cell–cell junction during cell adhesion process through an E-cadherin- and F-actin-dependent mechanism [22]. KLHL20 distribution to PML nuclear bodies is likely mediated by its interaction with PML, a key molecule for the assembly of this subnuclear domain. In response to signals that elevate the level of PML, such as IFN-α/γ, an enrichment of KLHL20 in PML-nuclear bodies is observed, which is accompanied with the decrease of its localization in cytoplasm [24]. Consequently, binding of KLHL20 to its cytoplasm substrate is reduced. Thus, alteration of subcellular localization is likely a mechanism for KLHL20 to selectively target a particular set of substrates under specialized physiological conditions. This provides an opportunity for KLHL20 to participate in diverse cellular processes at a highly regulated basis.

### Regulation of KLHL20 by KLHL39

In addition to regulating KLHL20 functions by altering its subcellular localizations, a recent study identified an inhibitory protein for KLHL20-based ubiquitin ligase. Interestingly, this inhibitor shares a similar domain structure with KLHL20 and is named KLHL39. Originally identified as a protein interacting with the NS1 protein of influenza A virus [95], KLHL39 was recovered from a yeast two-hybrid screen with the kelch-repeat domain of KLHL20 as bait [96]. Despite being recruited to the substrate-binding domain of KLHL20, KLHL39 is not a substrate of KLHL20-based Ubiquitin ligase. More surprisingly, KLHL39 does not bind Cul3 by carrying certain nonconserved residues in regions of its BTB domain that are equivalent to regions critical for contacting Cul3 in other BTB proteins. Subsequent analysis revealed that KLHL39 blocks KLHL20 binding to its substrates, such as DAPK and PML, implying that KLHL39 functions as a pseudosubstrate for KLHL20-based ubiquitin ligase. However, unlike several pseudosubstrates for the Cul3-Keap1 ligase, KLHL39 can additionally disrupt the formation of KLHL20-Cul3 complex with an unknown mechanism. Through these dual inhibitory roles, i.e., inhibition of KLHL20 binding to Cul3 and substrates, KLHL39 blocks KLHL20-dependent ubiquitination and degradation of DAPK and PML, leading to an increase of their steady-state levels. These findings uncover not only a novel mode of regulatory mechanism for Cul3 family ligases but also a hierarchy among KLHL family members. KLHL39 is likely evolved to divergent from the Cul3 substrate adaptor role of KLHL family to an inhibitory role for a particular KLHL member, thus increasing the functional versatility of this family.

Since DAPK and PML both act as tumor suppressors, the ability of KLHL39 to prevent their degradation predicts a similar function of KLHL39. In line with this notion, the murine ortholog of KLHL39, Nd1-L, is downregulated in metastatic lesions of colon cancers compared with the primary tumors and Nd1-L overexpression in a murine colon cancer cell line suppresses migration, invasion, and metastasis [97]. Similar tumor suppressive functions are observed with KLHL39 in human colon cancer cell lines, and more importantly, these functions are all mediated by a PML- and DAPK-dependent manner [96]. Clinical analysis revealed that low expression levels of KLHL39, DAPK, and PML are associated with metastatic progression of human colon cancers. These findings indicate a role of KLHL39-mediated PML and DAPK stabilization in colon cancer metastasis and imply an importance of KLHL20/KLHL39 ratio in determining the metastatic fate of tumor cells.

## Conclusion remarks

In this review, we summarize the diverse functions of KLHL20 and most of these functions are mediated by ubiquitination of a particular protein. We also discuss several regulatory mechanisms for KLHL20-based ubiquitin ligase, including the alterations of its expression and subcellular localizations, and the existence of an inhibitory protein. In addition to participating in many normal cellular processes, KLHL20 is regulated and elicits important roles in several stressed conditions, such as hypoxia and autophagy. Furthermore, KLHL20 is upregulated in certain tumors and possesses pleiotropic tumor-promoting functions through potentiating the degradation of tumor suppressor proteins DAPK and PML. KLHL20-based ubiquitin ligase is therefore a potential target of anti-cancer therapy.

Several important issues remain to be addressed. First, among the various KLHL20 substrates, it is unclear whether they carry a similar sequence motif, i.e., degron, for KLHL20 binding. Identification of KLHL20-specific degron would aid in the discovery of additional KLHL20 substrates. Second, KLHL20 is capable of catalyzing polyubiquitination with distinct chain specificities on different substrates, such as K33-linked chain on Crn7 and K48-linked chain on other substrates, but its underlying mechanism remains elusive. Third, KLHL20 knockout mice show a number of prominent defects but the precise molecular mechanisms are poorly characterized. It is also unclear whether these defects are related to insufficient ubiquitination of certain proteins. Answers to these questions would be helpful for our understanding of the functions and mechanisms of this versatile protein.
